# Supplementary material for: Are carbon emissions trading and green financial instruments synergistic? -Comprehensive quantitative research based on content analysis
Source: PLoS One. 2024 Mar 7;19(3):e0298601. doi: 10.1371/journal.pone.0298601 (PMC10919601; doi:10.1371/journal.pone.0298601)
Supplement: S1 Data — (ZIP) [file pone.0298601.s001.zip › Data/地级市Z_F工作报告.zip!/║╙▒▒/▓╫╓▌╩╨/└┤╫╘╣1⁄2╓┌║┼í╛┬φ┐╦╩2╛▌═°í┐_2003.docx]

各位代表：

现在，我代表沧州市人民政府，向大会作工作报告，请各位代表审议，并请各位政协委员和列席会议的同志提出意见。

过去五年政府工作的回顾

市十届人大一次会议以来的五年，是我市经济和社会发展的重要时期。在省委、省政府和市委的正确领导下，我们坚持以邓小平理论和“三个代表”重要思想为指导，紧紧依靠全市人民，团结奋斗，开拓创新，圆满完成了市十届人大各次会议确定的主要任务，经济和社会各项事业取得了新的成就。

一、国民经济保持了较快的发展速度。2002年，全市国内生产总值实现531.3亿元，是1997年的1.7倍，年均增长10.6％。全部财政收入达到52.98亿元，其中，市县级一般预算收入21.4亿元，年均分别增长22.8％和16.2％。

农业和农村经济结构调整取得明显成效。以推进农业产业化为重点，大力实施“四大产业、一大工程”，在连续干旱的情况下，农业保持了稳定发展，产业化水平进一步提高。2002年，全市枣草畜菜四大产业实现产值79.6亿元，占农业总产值的53.1％，比1997年增长31.3％。农村非农产业发展较快，2002年，全市乡镇企业完成增加值304.9亿元，比1997年增长146.4％，成为最具活力的经济增长点。

工业经济运行质量逐步提高。五年来，共完成技改投资71.8亿元，相继完成了大化2万吨TDI、沧化23万吨PVC、沧炼“735”改造、东塑聚乙烯燃气管材管件等一大批新建和技改项目，启动建设了临港化工园区。完成市级以上新产品开发420项，其中有60项处于国内领先水平。2002年，全市规模企业完成增加值77.6亿元，利税23亿元，与1997年相比，年平均递增11.2％和5％。

服务业发展步伐加快。市场建设遥遥领先，2002年完成交易额141亿元。引进先进经营管理模式，创新经营业态，传统商贸流通业在竞争中不断发展壮大。房地产、非义务教育、社区服务、旅游业、信息产业和中介服务等现代服务业迅速发展，逐步成为经济增长的新亮点。

二、投资对经济增长的拉动力明显增强。五年来，全市累计完成固定资产投资678.6亿元，年均增长8％。完成投资1000万元以上的重点建设项目206个。其中，完成黄骅大港一期、朔黄铁路、京沪与石黄高速公路、改水降氟二期、天然气管网建设等重大基础设施项目68个；完成东塑有机硅表面活性剂、宝丰超高压线缆、振海集团高档铝合金门窗等生产性项目105个；完成孟村宏达肉牛深加工、沧县崔尔庄红枣批发市场等农业产业化龙头项目21个，为经济稳定增长积蓄了后劲。

大力加强城市基础设施建设和综合整治，组织实施了街道拓宽、延伸和广场建设，集中供水、供气、供热，以及通透绿化、净化美化等一批重点工程，街路环境和城区容貌得到较大改观。经营城市取得新进展。2002年，全市收取城市土地使用权出让金9亿多元。

三、改革开放步伐进一步加快。企业改革不断深化。全市3604家乡及乡以上国有集体企业中，83.9％已完成产权制度改革，有30家大中型企业建立了比较规范的现代企业制度。“帮危解困”工程取得明显成效。全市工业系统142家危困企业已有89家退出危困行列。政府机构改革基本完成，行政审批制度改革迈出坚实步伐。新的财政分税制体制平稳运行，农村税费改革进展顺利，农民负担得到不同程度的减轻。积极做好防范和化解财政金融风险工作。通过增收节支，消化市本级历年财政赤字6000多万元，农村合作基金会得到全面清理，新华区债券问题得到初步解决。社会保障体系日趋完善。基本建立了以城镇养老、失业、医疗保险等为主要内容的社会保障体系，实现了两个“确保”。

对外开放不断扩大。五年来，全市实际利用外资累计3.81亿美元，其中直接利用外资1.36亿美元。2002年，成功举办了沧州—韩国、沧州—日本中小企业合作恳谈会，承办了中韩联合发行的《武术与跆拳道》邮票首发式，实现了自办国际性招商活动“零的突破”。与104个国家和地区建立了经贸关系，累计实现对外出口7.7亿美元，年均递增19.3％。对内开放进一步加快。累计签定经济合作项目756项，引进资金25.37亿元；

四、社会事业全面发展。五年来，全市取得市级以上科技成果297项，科技成果推广应用率由1997年的67％提高到2002年的78％，高新技术技工贸总收入达到36.5亿元，年均增长45.5％。教育事业在发展中壮大。五年间，累计投入资金11.1亿元用于改善办学条件，消灭危险校舍24175间。基本普及了九年义务教育，小学、初中在校生巩固率分别达到99.8％和98％。高等教育、职业技术教育及幼儿教育有了新的发展。文化事业繁荣活跃，卫生、体育、广播电视、统计、审计、物价、人防、民族宗教、残联、老龄、档案等工作都取得了新的成绩。

可持续发展能力不断增强。五年来，全市计划生育率始终保持在91％以上，人口出生率稳定在11‰以下。完成了，国土资源总体规划，实现了耕地占补平衡有余。累计投入污染源治理资金11.2亿元，对1270家重点工业企业进行了治理。

五、精神文明建设和民主法制建设进一步加强。坚持用邓小平理论和“三个代表”重要思想武装干部群众，群众性精神文明创建活动取得明显成效。大力开展反腐倡廉教育和警示教育，讲团结、树正气，基本消除了原市委主要负责人腐败案件的消极影响。社区建设、基层民主政治建设扎实推进，政务、村务、厂务、校务公开继续扩大。民兵、预备役建设和国防动员工作得到加强，军民共建、拥政爱民、拥军优属活动进一步开展。各级政府自觉接受同级人民代表大会及其常委会的监督，充分听取政协和各民主党派、工商联、无党派人士及专家学者、人民群众的意见和建议，积极支持工会、共青团、妇联、科协等人民团体开展工作。积极稳妥地处理了12．12事件，保持了社会稳定。深入持久地开展“严打”斗争和社会治安综合治理，依法打击“法轮功”等邪教组织，认真落实人民内部矛盾纠纷排查调处制度，及时妥善处理群体事件和信访问题，社会秩序和社会风气进一步好转。

六、人民生活水平逐年提高。坚持把提高和改善群众生活作为政府的重要职责，通过发展经济不断改善和提高群众生活水平。五年来，王大引水、改水降氟、人畜饮水、广场道路、安居住房、垃圾处理、天然气入户、有线电视等一大批与群众生产生活息息相关的工程项目相继完工并投入使用。累计为全市城镇保障对象按时足额发放最低生活保障金3452万元，为农村25.43万户灾民发放救济款4459万元。帮助全市32715名国有和集体企业下岗职工实现了再就业。2002年，全市城镇居民人均可支配收入和农民人均纯收入分别达到6150元和2549元，比1997年增长19.98％和11.36％；城乡居民储蓄存款余额由1997年的275.14亿元增加到487.06亿元。

五年的发展实践使我们深深地体会到：第一，必须紧紧抓住发展这个“第一要务”，聚精会神搞建设，一心一意谋发展。面对前进中的各种矛盾和问题，只有坚持“发展是硬道理”的思想，用发展的办法解决前进中的问题，不断解放和发展生产力，才能实现经济社会的不断进步；第二，必须坚持解放思想，实事求是，与时俱进，创造性地开展工作。只有善于分析形势、把握机遇，不断地解放思想和改革创新，才能准确把握时代发展的主旋律，冲破旧观念和旧体制的束缚，不失时机地推动全市经济社会加速发展；第三，必须坚持执政为民，始终代表人民群众的根本利益。只有紧紧依靠人民群众，把关心群众作为一切工作的出发点和落脚点，时刻把握群众脉搏，维护最广大人民群众的根本利益，才能凝聚人心，鼓舞士气，形成加快发展的强大合力；第四，必须正确处理改革、发展、稳定的关系，在社会政治稳定中推进改革和发展，在改革发展中保持社会稳定。改革是发展的动力，稳定是改革和发展的基本前提，只有正确处理好三者之间的关系，才能集中精力谋发展；第五，必须求真务实，突出重点，狠抓工作落实。只有坚持一切从实际出发，大力弘扬脚踏实地、求真务实的优良作风，抓重点、攻难点，才能保证决策部署的贯彻落实和各项任务目标的顺利实现。几年来的发展实践昭示我们，只要我们坚定不移地贯彻“三个代表”重要思想，紧紧抓住发展主题，与时代发展同步伐，与人民群众心连心，沧州的未来就充满希望。

各位代表，我们所取得的各项成绩，凝聚着全市人民的心血和汗水，体现了方方面面的帮助和支持。在此，我谨代表沧州市人民政府，向辛勤工作在各个领域和岗位上的全市人民，向给予我们支持与监督的人大代表、政协委员，各民主党派、工商联、无党派人士和各人民团体，向大力支持我们工作的驻沧单位、人民解放军和武警部队，向海内外的在沧投资者，以及所有关心支持沧州发展的各界人士，表示崇高的敬意和衷心的感谢!

在充分肯定成绩的同时，我们也清醒地认识到前进中的困难和问题，主要是：农业产业化龙头带动作用还不强，农业综合生产效益较低，农民增收步伐缓慢；工业主导产业单一，高新技术产业比重较小；城市化水平不高，城乡经济发展不够协调，部分县乡财政增长乏力；对外开放水平不高，利用外资规模偏小；一些政府部门工作人员思想仍然不够解放，中心意识、大局意识、服务意识不强，经济发展环境还不够宽松。对于这些问题，我们将采取有力措施，认真加以解决。

今后五年的发展目标和战略举措

各位代表，新一届政府将以邓小平理论和“三个代表”重要思想为指导，全面贯彻党的十六大精神，树正气、讲团结、求发展，紧紧围绕全面建设小康社会的总体目标，加快调整和优化经济结构，大力推进体制创新和科技创新，加速新型工业化和城市化进程，着力壮大工业经济，积极发展农村经济，协调发展城乡经济，全面提升服务业整体水平，努力把沧州建设成为国内重要的港口贸易通道、有竞争力的化工产销基地、特色突出的绿色农业基地，为全面建设小康社会奠定坚实基础。

今后五年的发展目标是：

——经济发展再上新台阶。力争“十五”后3年，全市经济增长年均达到9.5％以上，在确保完成“十五”计划的基础上，到2007年，国内生产总值达到880亿元左右，人均国内生产总值按现行汇率折算达到1540美元。经济结构调整取得显著成效，经济、社会和环境协调发展。

——城乡面貌出现新变化。加快城乡基础设施建设、生态环境建设和人文景观建设。城市污水处理率达到60％以上，生活垃圾无害化处理率80％以上，绿地率、人均公共绿地面积和绿化覆盖率有较大幅度的提高。城市化水平到35％。

——人民生活得到新改善。到2007年，全市城市居民人均可支配收入达到7850元，农村居民人均纯收入3100元；人均道路14平方米，人均住房建筑面积达到30平方米。人民生活更加富裕。

——社会文明实现新进步。公民思想道德素质、科学文化素质、身体健康素质和民主法制意识有较大提高。“普九”成果全面巩固，义务教育均衡发展。基本普及高中阶段教育，提高高等教育入学率。文化、卫生、体育、广播电视等社会各项事业有较大发展。

为实现上述目标，我们必须做到：

一、把握机遇，迎接挑战，咬定发展不放松。今后五年，我们面临着重要的发展机遇，从国际国内的大背景来看：经济全球化促进了各种生产要素在世界范围加速流动，为我们全方位扩大开放创造了条件；党的十六大制定了全面建设小康社会的宏伟蓝图，实现中华民族伟大复兴的序幕已经拉开；国家继续坚持扩大内需的方针，实施积极的财政政策和稳健的货币政策等等，为我们加快发展带来了新的机遇。就我们沧州来说：经过20多年的改革开放，特别是经过前五年的艰苦奋战，我们为更快发展积累了较为丰厚的物质基础和综合实力；黄骅大港的建成，临港化工园区的启动等等，更为沧州的发展提供了强大的拉动力；面对这些客观条件和物质基础，再加上全市人民想发展、盼发展的高涨热情，我们可以断言：沧州应该发展快一些，沧州有条件发展快一些!在看到机遇的同时，我们也应清醒地看到所面临的前所未有的挑战：我国加入世贸组织后，我们的政策环境还不适应与国际通行规则接轨的要求；随着国家在更多领域，更深层次的开放和外资的不断涌人，我们现有的产业、技术和管理将不可避免地受到冲击；全国正在掀起新一轮的改革发展热潮，区域经济竞争日益激烈。举国上下，东西南北，大家都看到了发展机遇，并且都在抢抓这一机遇。面临这样的挑战，不进则退，慢进也是退，不思进取会失误，动作迟缓同样会失误，机遇和挑战从两个侧面给予我们同一个启示：必须象打仗抢占制高点一样，迅速行动，抢占发展先机。这就要求我们以更加强烈的忧患意识，更加积极的进取姿态，更加集中的精力和更加务实的举措，紧紧抓住本世纪头20年这个重要的战略机遇期，扭住发展不放松，开创我市经济和社会各项事业大发展、快发展的新局面。

二、统一思想，坚定信念，迅速驶入快车道。我们要想快速健康发展，首先必须在四个方面统一全市人民的思想，形成共识。

工业强市。毫无疑问，农业、农村、农民仍是我们全市工作的大头。但是，几十年的经验证明，如果只是就“三农”讲“三农”，永远也解决不了农村和农民问题，永远也建不成经济强市。因此，必须遵循党的十六大精神，加快工业化进程，以工立市，以工兴市，以工强市，迅速提高工业经济对GDP、城乡居民收入、财政收入、社会就业、农村人口转移的贡献率。坚持城乡工业协调发展，大力发展城乡互补型精深加工业。充分发挥后发优势，以信息化带动产业化，走新型工业化道路，用工业化、信息化把沧州推上现代化建设快车道。

项目拉动。上工业必须有项目作为载体，抓经济必须把项目作为切人点，求发展必须用项目搭建平台，增投入必须靠项目牵引拉动。总而言之，聚精会神搞建设，没有项目，建设无从谈起，一心一意谋发展，没有项目，发展无从抓起。我们要加快发展，以快赶强，必须树立强烈的项目意识，真正谋划、上马一批足以立市的新项目、大项目。

营造经济特色。面对日趋激烈的竞争，我们要清醒地看到，作为历史上的欠发达地区，我们本来就和竞争对手不在同一条起跑线上，在这种情况下，我们还想赶上甚至超过对手，就必须营造经济特色。无论是工业还是农业，是生产还是流通，没有特色不仅难以做大做强，尤其难以经久不衰。对于经济工作来说，特色就是优势，特色就是品牌，特色就是在竞争中克敌制胜的法宝。要善于品牌运作，不断推出新的特色产品。在尽快形成一批优势特色工业产业的同时，大力培育特色高效农业，尤其要下大力打造出沧州红枣和苜蓿的品牌。

时刻不忘“三农”。我们主张靠实现工业化从根本上解决“三农”问题，主张解决农民问题的根本出路在于推进城镇化，发展非农产业，减少农民数量。但是，我们必须看到，这需要一个过程，在相当长的一个时期内，农业、农村、农民仍然是我们的主要工作对象。“三农”问题仍然是新一届政府面临的突出问题。我们必须全面贯彻执行党在农村的各项方针政策，增加对农业的投入，改善农业生产条件，加快农业产业化进程，千方百计增加农民收入，为从根本上解决“三农”问题积累条件，夯实基础。

三、理清思路，突出重点，力争有所作为。经济工作千头万绪，有所不为才能有所为。因此，必须找到自己的优势所在，扬长避短；必须不断开拓新的经济增长空间，增育新的增长极；必须善于借助外力，给自己插上一对腾飞的翅膀。首先，扩大对外开放。要看到在全球经济一体化踏步前进的时候，作为一个地区，不是想不想开放的问题，而是必须开放。改革开放20多年的实践证明，对外开放的程度，决定着经济发展的速度。我们要充分发挥“两环”区位优势和沿海临港的客观条件，主动融入世界经济大循环，积极参与首都经济圈经济技术分工与协作，切实改变招商方式，扩大利用外资领域，不断提高经济外向度，千方百计形成与沧州作为沿海城市相称的对外开放新格局。其次，发展民营经济。在发展民营经济方面，我们已积累了比较丰富的经验，尤其是过去五年的实践证明，民营经济已经成为我市独具发展优势的经济增长点。今后五年我们要继续毫不动摇地发展民营经济，县及县以下不再兴办国有独资企业，全市所有允许外资进入的领域全部向民营经济开放。要尽快制定完善相应的政策，创造良好的发展环境，让民营经济不仅能够生根发芽，而且能够长成参天大树。第三，培育临港产业。黄骅大港是我们独特的优势，世界上以港兴市、以港兴业的成功范例屡见不鲜。我们拥有全省近l／4的海岸线，过去我们常常为沿海上百万亩寸草不生的盐碱地而悲凉，为没有一寸可供开展旅游的沙滩而遗憾。今天，当大港建成，铁路开通，当临港化工园区和神华万亩工业园纷纷启动之时，我们才发现，这上百万亩的滩涂荒地，对于工业开发竟是得天独厚的土地资源，我们一定要放开手脚乘势而上，把码头、化工、远洋捕捞和出口加工等这些临港沿海的产业做大做强，真正把区位优势，资源优势变为经济优势、产业优势。第四，加快城镇建设。工业化是城镇化的支撑，而城镇化又是工业化的载体。要实现现代化必须实现城镇化，城镇化是全面建设小康社会、实现现代化的必由之路要采取切实措施，加速沧州市区的规模扩张和人口膨胀。同时以产业为依托，大力推动小城镇建设，逐步形成以沧州市区为中心，以港城、县（市）城和中心城镇为支点，布局合理、协调发展的现代化城镇体系。要打破马路加楼房就是城市的陈旧思维，坚持以人为本加强城市建设，立足于建设生态城市、园林城市，构建城市特色。

四、讲究策略，采取措施，保障重点工作顺利实施。为了各项战略重点得以实施，还必须讲究策略，采取切实措施予以有力保障。首先，坚持科教兴市。着眼于增强科技创新能力和提高人口素质，加大科教投入，深化科教体制改革，提高科学技术对经济和社会发展的贡献率，鼓励技术革新和新产品开发，健全科技推广体系，加快科技成果向现实生产力的转化。同时努力推进教育结构调整，优化学校布局和教育资源配置。其次，不断改革创新。创新是一个民族进步的灵魂。同样，创新也是一个地区进步的灵魂。不论是经济建设还是精神文明建设，都必须树立强烈的改革意识和创新理念，不断改革，不断创新，不断赋予我们的事业以生机和活力。由于处在体制转型期，我们面临着许多困难和问题，企业经营困难、劳动力就业困难等等，要解决这些困难和问题，指望旧体制复归是不可能的，必须大力度推进改革，向改革要出路，向改革要发展；面对不断出现的新情况、新课题、新挑战，以不变应万变是不行的，必须大胆创新方能出奇制胜。因此，务必要做到发展要有新思路，改革要有新突破，开放要有新局面，各项工作要有新举措。第三，优化发展环境。经济发展有许多制约因素，但环境问题越来越成为普遍关注的焦点，越来越成为直接影响经济发展的要素。在交通、区位、招商政策等条件日趋相近的情况下，谁的发展环境好，谁就对人才、资金和项目等具有更强的吸引力和凝聚力。没有一个好的发展环境，就没有沧州的大发展。这一点必须成为全市人民的共识，进一步强化抓环境就是抓竞争，抓环境就是抓发展的意识，大力优化经济发展环境。第四，确保社会稳定。实践证明，有了稳定的社会环境我们的事业才能够蓬勃发展，我们的人民才能够安居乐业。因此，必须正确处理改革、发展、稳定的关系，时刻牢记稳定压倒一切，每个人都象珍惜自己的眼睛一样，珍惜我们的安定团结，珍惜我们的社会稳定，让沧州的经济列车快速平稳地奔驰在发展的轨道上。

2003年政府工作的重点

今年国民经济和社会发展的主要预期目标是：国内生产总值比上年增长10％。全社会固定资产投资增长10％以上。全部财政收入增长8％以上。城镇居民人均可支配收入和农民人均纯收入分别增长3％左右。

第一季度全市经济运行情况良好，GDP同比增长13％，全社会固定资产投资同比增长143.7％，直接利用外资同比增长8.7％，地方财政收入同比增长20％。如此良好的开局令人欣喜，令人振奋。正当全市人民群情激昂、高歌猛进之时，非典型肺炎突如其来，使我们同全国人民一样经受了一场灾难。一时之间，抗击“非典”，确保人民生命安全，成了全市各项工作的重中之重。万众一心，和衷共济，经过几十个昼夜的艰苦奋战，终于取得了抗击“非典”斗争的重大胜利。但是，“非典”已经给我们正常的工作和生活秩序造成严重影响，给我们的经济工作带来意想不到的困难。本届人大第一次会议也不得不因抗击“非典”而延期召开。当大会开幕之时，全年已经时间过半。因此，新一届政府的开局之年，只剩下半年。但是，我们要奋力拼打这半年，把因“非典”造成的损失补回来，誓夺全年任务目标的圆满实现。

一、以结构调整为切人点，千方百计地增加农民收入。一是加强优质高效农产品产销基地建设，推动“四大产业、一大工程”扩规提质增效。重点抓好60个农业结构调整示范乡、360个示范村，以及8条主干道两侧特色高效农、林、牧示范走廊建设；抓好泊头、黄骅、沧县、献县等梨枣生产基地，青县、肃宁等七个蔬菜生产基地建设。充分利用国家退耕还林政策，全面完成40万亩速生丰产林、30万亩红枣基地建设任务。年内新增苜蓿种子田l万亩，优种率达到98％以上。依托饲草资源，加快发展畜牧养殖业。二是大力发展农业产业化经营。重点扶持黄骅三利、沧州沛然世纪、任丘大发正大、中捷乡谣等50家农业产业化龙头企业，扩大规模，提升档次，增强辐射带动能力。加强农业行业协会和中介组织建设，充分发挥市草业、枣业协会的作用，提高农民的市场化组织程度。三是积极发展非农产业。积极引导和鼓励农民向农产品加工、流通和农业服务等行业转移，规范有序地组织好民工进城和劳务输出，拓宽农民增收渠道。四是加大对农业的投入。用足用好国家政策，搞好开发式扶贫。鼓励多元资本以各种形式投资农业和水、电、路、桥等农村基础设施建设，改善农业生产条件和农民生活。

二、加大投入，优化结构，提高工业经济的竞争力和发展后劲。切实强化工业强市思想，牢固树立大抓工业、抓大工业的强烈意识，加大投资力度，加快项目建设，拉动工业攀升。要运用市场经济机制，抓好项目建设中的谋划、跑办、融资三个关键环节。立足资源和产业优势，开阔视野，谋划项目；不辞辛苦，善于变通，以坚韧不拔和攻坚克难的精神跑办项目；用足用活上级政策，搞好资本运营，拓宽融资渠道，千方百计保证重点项目资金需求。全力支持朔黄铁路复线、黄骅港二期、沧东电厂一期、黄骅港杂货码头等国家和省重点项目建设。力争沧县远东高密度互联印刷电路板、沧州中药厂年产21亿粒唐威胶囊、盐山千童酒业枣膳食纤维素等20个申报待批大项目取得突破性进展。确保大化三聚氯胺、盐山无缝化钢管、沧县红枣批发市场三期等92个投资1000万元以上的在建项目按期投产达效；沧化集团液体化学品码头、沧州运东污水处理厂等56个投资1000万元以上的新开工项目按期开工建设。

进一步优化工业结构，培育主导产业，提高工业经济的运行质量和经济效益。一是加快改造和提升传统产业。着力抓好沧炼1万吨聚丙烯专用料、东塑大口径波纹管、鑫源泰10万吨直缝焊管、泊头宏业集团汽车加热器等100项技改项目和100项新产品开发项目，确保完成工业技改投资15亿元以上。二是做大做强化工主导产业。大力推进临港化工园区建设，在加快基础设施建设的同时，重点加强与中石化、中石油、中海油等大企业的联系与合作，搞好总投资130亿元、28个大项目的推介发布，力争在招商引资上实现大的突破。依托临港化工园区和沧化、大化、沧炼三大化工集团，突出抓好大化5万吨TDI、沧化40万吨PVC、8万吨离子膜烧碱、沧炼油品升级改造等重大项目，搞好PVC、TDI、丙烯酸等产品系列开发。三是积极发展新兴产业和高新技术产品。着力培育和发展电子信息、生物制药、新型建材和环保卉业，逐步走出一条科技含量高、经济效益好、资源消耗低、环境污染少、人力资源优势得到充分发挥的新型工业化路子。

三、大力发展现代服务业，积极推进城镇化进程。努力培育生产要素市场，推动产权、土地、技术、劳动力等生产要素规范有序流动和优化配置。建立和完善金融、保险、中介、咨询、法律服务等经济保障体系，提高各类生产要素的市场化程度。加快发展信息服务业和社区服务体系。完善物业管理、信息网络、卫生医疗、连锁超市、文化娱乐、体育休闲等配套服务设施，推进社区服务社会化、产业化、网络化。改造提升商贸流通业，推动粮油配送中心等物流企业建设，培育大型商贸集团。积极发展旅游业，重点抓好吴桥杂技大世界、任丘白洋淀、海兴小山和湿地保护区、民间武术等旅游项目开发，推动旅游产业化。

第三产业的发展尤其需要以城镇化为依托。因此，要积极推进城镇化进程。年内完成沧州新区规划，启动新区基础设施建设，逐步把新区建成行政、文体、教育租会展中心。要精心设计，追求特色，引水入城，突出绿色。将来每一个来沧州的人一下公路、一出车站、一进沧州，便能感受到强烈的滨海城市风格和现代化都市气派。同时，抓紧现有城区的改造，抓好市区给排水、绿化、亮化、污水和垃圾处理等重点工程。建好鼓楼广场、胜利广场和小南门步行街，谋划高水准的运河景观带，综合整治市区环境建设的突出问题。立足城乡统筹，力求合理布局，积极发展中小城镇。改革户籍管理，降低进城门槛，使各个县（市）城加速膨胀，充分利用城镇的聚集效应，使服务业得到空前繁荣。

四、以体制和机制创新为重点，不断完善社会主义市场经济。按照市场经济的要求，积极引导国有资本合理流动，以产权制度改革为重点，继续推行股份制和股份合作制改造，积极发展混合所有制经济。重点抓好沧化集团、沧运集团、东塑集团的低成本扩张，以及市属商贸流通系统33家未改制企业的整合改组、改造，逐步做到股权多元化、经营市场化，管理现代化。深化分配制度改革，鼓励和支持劳动资本、技术管理等生产要素按贡献参与分配。全面推进以国有粮食企业产权制度改革为重点的粮食流通体制改革。整顿和规范市场经济秩序，依法严厉打击制售假冒伪劣商品、偷税骗税和价格欺诈等不法行为。继续深化预算外资金“收支两条线”改革，确保预算外收入及时足额收缴到位。巩固农村税费改革成果。加快国有资产管理体制改革，大力度盘活存量资产，确保国有资产保值增值。加强财政和金融监管，防范和化解财政金融风险。深化社会保障体制改革，完善配套政策，扩大覆盖面。健全和完善失业保险制度和城市居民最低生活保障制度。进一步搞好医药卫生行业三项制度改革试点，做好重大疫情的预防和紧急处置工作。积极拓展就业渠道，开辟新的就业岗位和领域，努力扩大就业和再就业。

五、以扩大利用外资为重点，力求对外开放取得新突破。坚持把利用外资放在首位，精心筛选招商项目，精心组织招商工作，不务虚名、不图形式，减少盲目出国、参展、赴会，积极谋划网上招商，努力提高招商的成功率。重点抓好关联度大、技术水平高的大项目和对世界500强及其他知名大企业的招商工作。加大国内社会和民间资本的招商引资工作力度，努力促成新一轮招商引资热潮。努力扩大出口，壮大出口企业队伍，优化出口产品结构，巩固和拓展国际市场。大力改进小枣、裘皮、服装、抽纱等优势产品的生产工艺，提高质量档次，扩大出口创汇。同时，积极实施“走出去”战略，鼓励有实力的企业到境外投资兴业，鼓励扩大境外工程承包和劳务合作规模。总之，务求对外开放有所突破。

六、营造良好环境，推动民营经济快速发展。要努力创造促进民营经济发展的良好环境和浓厚氛围，使民营企业创业人员社会上有地位，政治上有荣誉，经济上有实惠，以促进民营经济更快更好发展。积极鼓励和支持国有集体企业改制一批，骨干民营企业膨胀一批，市外民营企业引进一批，广泛发动发展一批，不断壮大民营企业队伍。加强对民营企业的引导和服务，鼓励和支持有实力、有条件的民营企业参与国有企业的战略性重组，实现投资主体多元化。鼓励和引导民间资本投向能源、交通、供水、环保等基础设施及公用事业建设。要特别注重培育壮大民营经济中的特色优势行业，在电线电缆、裘皮制革、电子五金、轻工塑料和食品等特色行业中选择一批优势企业，予以重点扶持。同时，积极推动民营企业向园区集中，推动民营企业园区向特色园区、专业园区和科技园区发展。

七、开发新的经济增长区域，壮大新的经济增长点。一是理清思路，制订规划，发展临港产业和海洋经济。年内要召开海洋经济发展研讨会，以新的视角理清海洋经济的内涵，制订出符合沧州实际的海洋经济发展战略，把海洋经济作为我市经济腾飞的一个重要支点谋划好、运作好。对于传统的和比较成熟的利用海洋资源的产业和项目，譬如海水养殖和远洋捕捞、浅海滩涂开发、海产品保鲜和精深加工等，要努力扩大规模，强化装备，采用先进技术，不断提高产量和效益。同时，积极发展临港产业，依托港口，面向腹地和周边地区，积极发展中转型内外贸易，以及与港口配套的仓储业、运输业。二是努力开拓新的经济增长区域。沧州经济技术开发区、黄骅港开发区、临港化工园区和沧州新城区，是经过多方筹划、反复论证、普遍看好的新的经济增长区域。这四个区域优势明显，特色分明，是为全市经济快速发展搭建的新平台。尚未启动的，年内要举全市之力，促其启动；已经启动的，要千方百计加速运转。同时，努力抓好各县（市、区）所属的开发区和园区建设，理顺管理体制，加快发展步伐。

八、加强精神文明建设，推进社会各项事业全面进步。坚持物质文明、政治文明和精神文明建设同步推进，努力实现经济与社会各项事业协调发展。

大力推进民主法制建设。自觉接受人大及其常委会的监督，支持人民政协发挥政治协商、民主监督和参政议政职能，及时办理人大代表、政协委员的各项议案、建议和提案。加强与各民主党派、工会、共青团、妇联等人民团体的联系，虚心听取社会各界人士的意见和建议。进一步推进政务、厂务、村务和校务公开，不断扩大基层民主。切实加强思想道德建设。深入开展精神文明创建活动，认真贯彻《公民道德建设实施纲要》，加强社会公德、职业道德和家庭美德教育，全力打造“诚信沧州”，努力营造健康向上的社会风气。

发展科技教育，促进可持续发展。建立健全科技推广体系，继续实施农业“种子工程”，抓好小麦高产优质低耗栽培、果品嫁接改造、牛羊胚胎移植等实用技术的推广，不断提高科技的贡献率。加强基础教育，巩固提高“普九”成果，调整中等职业教育的办学思路，强化技能培训。扩大普通高中招生规模。积极运作沧州师专、河北工专升本及卫校、泊师、青县幼师升专工作。建立健全人口与计划生育工作管理新机制，由社会控制制约为主向依法管理、优质服务、利益导向转变，合理控制人口增长。进一步实施“碧水蓝天”工程，加强水、土等资源保护和治理工作，全面改善城乡生态环境。积极发展循环经济和绿色产业、逐步建立低消耗、可循环利用的绿色生产、生活和消费方式。大力开展植树造林，加强生态建设。下决心退耕还林，大面积推广林粮间作。从城市到乡村，人人动手，见缝插绿，让该绿的地方都绿起来，让能绿的地方都绿起来。

全面推进社会各项事业。加强农村和城市社区卫生服务，健全市、县、乡、村四级疾病防控体系。广泛开展群众性体育活动。加快广播文化基础设施建设，大力发展文化事业和文化产业。深入开展国防教育，加强国防动员、民兵、预备役和人防工作，搞好拥军优属、优抚安置和军民共建活动。认真做好民政、审计、物价、气象、地震、档案等各种工作。加强社会治安综合治理，依法严厉打击各种犯罪活动，防范和惩治“法轮功”等邪教组织，扫除社会丑恶现象。做好救灾救济工作，重视群众来信来访，开展民间纠纷集中排查，及时化解各种不稳定因素。抓好安全生产，消除事故隐患，确保人民群众生命财产安全。依法管理宗教事务，扶持民族经济发展，促进民族团结和社会进步。

九、转变政府职能，建设廉洁高效的人民政府。第一，树立政府就是服务的观念，建设高效服务的政府。新一届政府要继续转变职能，深化行政体制改革，推进政企分开、政事分开、政社分开，把该管的事管好，不该管的事坚决不管。其中，最该管的事就是为经济建设提供快速发展、公平竞争的良好环境。新一届政府要进一步精简审批项目，简化审批程序，提高工作效率，降低行政成本。进一步加强各职能部门为企业服务的协调配合，为企业提供全程服务，高效服务。痛下决心治理发展环境，大力推进行风建设，深入开展服务窗口竞赛和民主评议行风活动。在全市实行环境建设一票否决，让每一个政府部门和每一个公务员明白，为企业服务是自己的本职，服务的好是应该的，服务的不好就是过错、就是失职。第二，牢记两个“务必”，坚持廉洁从政。加强对政府工作人员，特别是各级领导干部的反腐倡廉教育，切实做到情为民系、权为民用、利为民谋。认真落实廉政建设责任制，加强对人、财、物管理使用的监督。强化预算管理，清理整顿行政事业性收费。抓好专项治理，严格查处“吃拿卡要”和“三乱”行为，严惩各类腐败分子。第三，不断解放思想，勇于开拓创新。要坚持党的解放思想、实事求是、与时俱进的思想路线。树立强烈的改革意识、创新意识和市场意识。自觉地把思想认识从那些不合时宜的观念、做法和体制的束缚中解放出来，一切以“三个有利于”为标准，以加快沧州发展为出发点和落脚点，敢闯敢试敢为天下先。第四，扎实工作，埋头苦干。新一届政府要唱响“树正气、讲团结、求发展”的主旋律，要有强烈的历史使命感和时代责任感，以实现沧州新一轮的发展为根本职责，忠于职守，奋发有为。要力戒官僚主义、形式主义，力戒摆花架子，作表面文章，求真务实，讲求实效，一切从实际出发。要深入基层，调查研究，体察民情，了解民意，满腔热情，认真负责地解决人民群众关心的热点、难点问题，倡导扎实肯干，少说多干，真抓实干，埋头苦干的作风。时刻牢记空谈误国事，实干兴沧州!各位代表，实现中华民族伟大复兴的号角已经吹响，沧州跨越式发展的目标已经确定，抓住这千载难逢的战略机遇，发展的快一些，就有希望跻身发达地区的行列，稍有失误延误了发展，就将给沧州带来历史的遗憾。让我们全市上下、万众一心，以党的十六大精神为指导，紧密团结在以胡锦涛同志为总书记的党中央周围，在中共沧州市委的正确领导下，咬定发展，务求必胜，为实现全面建设小康社会的宏伟蓝图，为造就更新更美更富强的沧州而努力奋斗！
